# Seroprevalence of toxoplasmosis and risk factors of *Toxoplasma gondii* infection among pregnant women in Sri Lanka: a cross sectional study

**DOI:** 10.1186/s12889-017-4941-0

**Published:** 2017-12-04

**Authors:** Devika Iddawela, Sanura Malinda Pallegoda Vithana, Chathura Ratnayake

**Affiliations:** 1 Department of Parasitology, Faculty of Medicine, University of Peradeniya, Peradeniya, Sri Lanka; 2 Department of Obstetrics and Gynaecology, Faculty of Medicine, University of Peradeniya, Peradeniya, Sri Lanka

**Keywords:** Toxoplasmosis, Sri Lanka, *Toxoplasma gondii*

## Abstract

**Background:**

*Toxoplasma gondii* is an intracellular protozoan infecting humans and animals. Infection in adults usually causes mild disease but greater importance lies in preventing transplacental transmission which can cause major foetal anomalies and is vital to identify infection in pregnancy. Research on this regard in Sri Lanka is scarce and would be beneficial in developing antenatal care strategies for improved foetal outcome.

**Methods:**

A random sample of 536 pregnant women attending antenatal care in Teaching Hospital Peradeniya from 2010 to 2013 was recruited for this study.

Blood samples were tested for *Toxoplasma gondii* IgG and IgM antibodies from the participants by using a commercial ELISA kit with a cut-off OD value of >1 and a structured questionnaire was used to identify the exposure to risk.

Bivariate analysis using the Chi Square test was used to calculate associations between documented risk factors and seropositivity and a *p* value of <0.05 was taken as significant.

**Results:**

Among the participants 160 (29.9%) were positive for *T. gondii* IgG antibodies and 2 (0.37%) were IgM positive. The seroprevalence in the first, second and third trimesters were 30.4%, 30.6% and 26.1% respectively. Of the risk factors studied, preparation and selling raw meat (*p* = 0.05) and household gardening (*p* = 0.01) were significant whereas the presence of domesticated cats and dogs, eating locally produced meat or dairy products did not show significant associations.

**Conclusions:**

Seroprevalence of *T. gondii* present among pregnant women attending antenatal care in Peradeniya was 29.9% indicating high level of transmission among the study population. However 70.1% of the study population were seronegative and were susceptible to primary acute infection during pregnancy and possible foetal anomalies. Therefore implementing health education especially on the aforementioned risk factors is recommended.

## Background

Toxoplasmosis is a zoonotic disease caused by an obligate intracellular organism, *Toxoplasma gondii (T. gondii)* which infects a vast range of warm blooded animals including humans. The definitive hosts are those belonging to the Felidae family. This parasite is transmitted to humans by the ingestion of food or water contaminated with oocysts shed by cats, eating raw or undercooked meat containing tissue cysts and by congenital transmission [1, 2]. Rarely recipients of blood and organ transplants can get infected if the donor is infected with *T. gondii* [3].

Acute infection in immunocompetent adults usually causes sub clinical infection. Symptomatic infection causes low grade fever, malaise, headache and cervical lymphadenopathy. Severe clinical manifestations such as encephalitis, myocarditis and pneumonia are rare and may even lead to death in the immunocompromised [4]. Latent infection may cause reactivation during pregnancy. This together with primary infection during pregnancy increases the risk of transplacental transmission. It is indeed the risk of congenital infection which is of greater importance [5].

Severity of congenital infection and the rate of transmission vary in different trimesters. The severity of infection is highest and the rate of transmission is lowest in the first trimester whereas the opposite is true for the third trimester [6]. Clinical presentations of congenital toxoplasmosis range from death in utero, severely affected infant to an infected but clinically unaffected child. The clinical spectrum may vary from clinically apparent birth defects such as hydrocephalus, microcephalus, and intrauterine growth retardation to subclinical infections leading to retinochoroiditis later in life [2, 7, 8].

Toxoplasmosis can be diagnosed by means of serology, culture based methods, mouse assays and PCR. The diagnostic accuracy increases when these methods are used in conjunction. *Toxoplasma* infection in pregnant women is typically diagnosed based on serology by detecting IgG, IgM antibodies and the avidity to *T. gondi* specific antibodies [9].

Global seroprevalence of toxoplasmosis ranges from 1% to 100% depending on a variety of factors: environmental, socioeconomic, food habits, healthcare facilities, hygiene and sanitation, host susceptibility, geographical location and soil humidity. Highest prevalence is seen in the humid regions of South America which is said to be over 90% while the lowest is in the Far East which is at 1% [10]. In the neighbouring country of India, seroprevalence was found to be 22.4% among women of childbearing age with wide geographical variation. A study conducted in Northeast India has concluded that the IgG seroprevalence among pregnant women was as high as 48% [11].

Sri Lanka is placed in the intermediate prevalence group (10% - 40%) amongst the Asian countries [12]. Two Sri Lankan studies carried out in the Colombo district, which is the financial and industrial capital of the country depicted seroprevalences of 22.5% and 27.5% among pregnant women [12]. In a study conducted in the district of Gampaha, neighbouring Colombo district, IgG and IgM seroprevalence among pregnant women was found to be 12.3% and 0% respectively [1]. No study has yet been carried out in the Kandy district which is situated in the central highlands of Sri Lanka to investigate toxoplasmosis in pregnant women.

The scarcity of studies done elsewhere in the country demands for more research to be done on *T. gondii* and toxoplasmosis due to its implication on pregnancy and congenital abnormalities. Our study aims to identify the overall seroprevalence of *T. gondii* and to identify the risk factors associated with it. This would be extremely useful in improving antenatal care in the region by implementing clinical and preventive strategies side by side.

## Method

### Study design and data collection

A cross sectional study design was used for the study. A total of 536 pregnant women attending antenatal clinic at the Teaching Hospital Peradeniya were randomly selected for the study. This hospital is a tertiary care centre in the island with a bed strength of 900. The hospital treats 70 K to 80 K in ward patients and 350 K out patients annually. About 600 deliveries are performed yearly in the Obstetric unit of this hospital. Data collection took place between 2010 and 2013 from pregnant women belonging to all 3 trimesters. An interviewer administered structured questionnaire with simple, closed ended questions was used to gather information regarding risk exposure such as contact with cats, consumption of undercooked meat/eating smoked meat/barbeque, working with raw meat at least once a week, household gardening. Contact with cats was defined as the presence of a cat at home for 3 months or more. This was also used to collect personal details of participants such as age, parity, period of gestation.

### Collection of blood

Ethical clearance for the study was obtained from the Ethical Review Committee of the Faculty of Medicine University of Peradeniya. Permission for data and sample collection was obtained from the Teaching Hospital Peradeniya. Study objectives, goals and procedure of sample collection were explained both verbally and in writing using native languages (Sinhala and Tamil) and informed written consent was obtained. Patient information and samples were kept confidential at all times. Samples were disposed appropriately after usage according to local policy.

Approximately 2 ml of venous blood was collected from each participant into a plain bottle (without anticoagulant) by venepuncture, using disposable syringes and needles under sterile conditions. Blood samples were then transported to the Department of Parasitology, Faculty of Medicine Peradeniya within 1-2 h. Serum was separated and was stored in labelled sterile containers at −20 °C until used.

### Serological testing

Testing for *Toxoplasma gondii* serology (IgM and IgG) was performed using commercial Enzyme Linked Immunosorbent assay (ELISA kit provided by Diagnostic Automation, INC USA which has a sensitivity and specificity of 94% and 100% respectively). Optical density (OD) values of >1 and >0.9 were taken as positive for IgG and IgM respectively.

### Data analysis

Data obtained was entered on to an excel spreadsheet and transferred to SPSS version 21 statistical program. Seroprevalence of *T. gondii* among the study population was calculated as the number of serologically positive subjects among the study group. Bivariate analysis using the Chi-square test was used to calculate the associations between the documented risk factors and seropositivity. Those with a *p* value <0.05 were considered significant.

## Results

Altogether, 536 pregnant women belonging to all 3 trimesters participated in the study. Among them, the majority (49.61%) were in the 2nd trimester followed by 33.9% and 16.4% in the 1st and 3rd trimesters respectively (Table 1). Out of 536 participants, 160 (29.9%) were positive for *T. gondii* IgG antibodies and only 2 (0.37%) of them were IgM antibody positive. The age distribution ranged from 16 to 56 with a mean age of 28.16 and a standard deviation of 5.45. Pregnant women who were <20 years showed the highest percentage (41.18%) of seropositivity whereas those >34 years had the lowest (Table 1). Age dependent seropositivity was not statistically significant. Of the total study population the majority (61.93%) were from rural households while the least number of participants (9.88%) came from semi-urban households (Table 1). Highest seropositivity was seen among those who resided in semi-urban households (39.6%). The IgG seroprevalence for the three trimesters were 30.4%, 30.6% and 26.1% respectively. Of the risk factors assessed, preparation or selling raw meat (*p* = 0.052,) and household gardening (*p* = 0.011) were identified as statistically significant risk factors associated with *Toxoplasma* IgG positivity. Although not statistically significant, antibody positivity was high (22.84%) among pregnant women who tasted meat curry during cooking. Large proportion (62.43%) of this study group did not have cats as household pets and of them 69.46% were seronegative. Of the pregnant women who owned household cats, the majority (71.14%) were seronegative and only 28.86% were positive for *Toxoplasma* antibodies. There was no significant association (*p* = 0.680) between cat ownership and *Toxoplasma* seropositivity (Table 2).

**Table 1:** Association of IgG Seroprevalence with demographic factors

Demographic factors	ELISA positive		ELISA negative			*p* value
	number	%	number	%	Total(*n*)	
• *Age group* (*n* = 536)						0.884
<20	7	41.18	10	58.82	17	
20-24	40	29.63	95	70.37	135	
25-29	52	29.71	123	70.29	175	
30-34	42	29.79	99	70.21	141	
>34	19	27.9	49	72.1	68	
Total	160	29.85	376	70.15	536	
• *Trimester* (*n* = 534)						0.716
First	55	30.39	126	69.61	181	
Second	81	30.57	184	69.43	265	
Third	23	26.13	65	73.87	88	
Total	159	29.78	375	70.22	534	
• *Parity* (*n* = 534)						0.59
Primi	71	28.06	182	71.94	253	
Multi	88	31.32	193	68.68	281	
Total	159	29.78	375	70.22	534	
• *Place of Residence* (*n* = 536)						0.196
Rural	92	27.71	240	72.29	332	
Semi Urban	21	39.62	32	60.38	53	
Urban	47	31.13	104	68.87	151	
Total	160	29.85	376	70.15	536

**Table 2:** IgG Seroprevalence with exposure related risk factors

Risk factor	ELISA positive	ELISA negative	Total	Chi square	*p* value
*Cats as pets* (*n* = 535)				0.17	0.68
Yes	58	143	201		
No	102	232	334		
*Dogs as pets* (*n* = 532)				2.654	0.103
Yes	72	201	273		
No	85	174	259		
*Consumption of undercooked meat* (*n* = 534)				0.887	0.346
Yes	14	43	57		
No	146	331	477		
*Tasting while cooking* (*at least once a week*) (*n* = 197)				2.045	0.449
Yes	45	59	104		
No	31	62	93		
*Consumption of local dairy/meat products* (*n* = 536)				0.573	0.431
Yes	3	4	7		
No	157	372	529		
*Raw meat handling in preparation or selling* (*n* = 533)				3.783	0.052
Yes	80	154	234		
No	79	220	299		
*Household gardening* (*n* = 536)				6.494	0.011
Yes	89	164	253		
No	71	212	283		
*Disease awareness* (*n* = 536)				0.031	0.860
Yes	36	82	118		
No	124	294	418		

## Discussion

The seroprevalence of *Toxoplasma gondii* in Sri Lanka has been classified as being moderate ranging from 10 to 40% [12]. The present study found that the overall seroprevalence of 29.9% to be higher than previous studies conducted in Sri Lanka, all of which were done in the Western Province which is primarily coastal and low lying in its geography [12]. Our study is the first study on *Toxoplasma* seroprevalence carried out in the Central Province which is geographically and socioculturally different than the rest of the country. In a study conducted in Northeast India, the highest *Toxoplasma* IgG positivity was found among pregnant women in hilly regions supporting our observation [11]. Oocyst survival mostly depend on environmental conditions. The moist conditions enhance the survival of oocysts for long periods and favour dissemination which could be the reason for high prevalence of toxoplasmosis reported in tropical countries [13]. The study population of this study was primarily from the wet and intermediate zones of the hill country where the temperature range from 17.5-30 °C throughout the year and receive 1250 mm to 3000 mm rainfall annually [14]. This facilitates sporulation and further development of oocysts to become infective.

The current study reported higher seroprevalence among participants from semi urban areas followed by urban and rural areas. Even though high prevalence have been documented from rural communities [15, 16], urbanization in developing countries is associated with poor socioeconomic conditions due to overcrowding and poverty resulting in higher seroprevalence [17, 18]. This suggests that the drivers of infection are far more complex than a simple urban-rural division and may be confounded by other factors.

In our study, *Toxoplasma gondii* IgM positivity was found in only 2 participants and all other seropositive cases were IgG positive indicating chronic infection. In agreement with our results, several studies have reported no or few IgM positivity compared to IgG [12, 19]. In patients with acute infection, *T. gondii* specific IgM appear initially and antibody levels become negative in few months. Persistent IgM positivity in chronic stages of *Toxoplasma* infection has been reported thus the presence of IgM antibodies does not always confirm acute infection [20]. However, negative *Toxoplasma* IgM test rules out recently acquired *Toxoplasma* infection [12]. This study reported a high percentage (70.1%) of seronegative pregnant women. This percentage could have been reduced if these seronegative women were followed up to delivery owing to possible seroconversion.

The age specific seropositivity was almost the same except for those who were under 20 years of age, which was very high at 41.2% (7 out of 17) whereas high IgG seronegativity was observed in older women (>34 years). But this difference was not statistically significant. A similar pattern was observed in studies done in Iran, Zambia and two local studies done in Colombo and Gampaha districts [21, 22]. High seropositivity among teenage pregnancies however was not seen in the local studies previously conducted in the island [1, 12]. Contrary to our results, a study in west Iran has observed high IgG seropositivity in older women (>30 years) compared to younger women (< 25 years) [23]. The high prevalence observed in younger participants in our study could be due to the lack of knowledge about the disease/pregnancy and greater exposure to risk factors. Apart from the risks of teenage pregnancy itself, the higher seropositivity seen in this age group demands education regarding toxoplasmosis and its consequences in antenatal clinics and in schools.

The first and second trimesters showed 30.4% and 30.6% seroprevalence while it was lower (26.1%) in the third but this was not statistically significant. Studies done in Colombo (Sri Lanka), Saudi Arabia and Zambia all showed IgG seropositivity to be highest in the 2nd trimester comparable to our results. Seropositivity in the third trimester even though being low in the present study, studies done elsewhere showed inconsistent values [12, 18, 22].

Among the exposure related risk factors analysed, handling raw meat during selling or preparation was identified as a significant risk factor associated with seropositivity. *T. gondii* have been isolated from tissues (brain, heart, muscles) of chicken in Sri Lanka and it has been well documented that beef, lamb and pork are good sources of *T. gondii* infection [24, 25]. The study population in the current study mainly comprised of housewives and they usually practiced cutting and washing meat before cooking. Therefore there could have been a high possibility of accidental ingestion of tissue cysts or tachyzoites from animal blood during meat handling. In a multicentre case control study conducted in Europe in 2000, tasting raw meat while cooking was found to have a significant association with *Toxoplasma* infection [26]. Tasting meat curry for flavour during and before cooking is practiced extensively in Sri Lankan households. Even though this was not identified as a significant risk factor for *Toxoplasma* infection in the present study, higher percentage of seropositives (22.84%) were reported among this group compared to those who did not taste meat curry during cooking which agrees with the European study. Our study did not identify eating raw or undercooked meat as a significant risk factor and previous studies conducted in Sri Lanka and Thailand have obtained similar results [7, 12]. Contrary to this, studies conducted in Cameroon, Japan and England have identified a significant association between consumption of raw and undercooked meat and *T. gondii* seropositivity [27–29].Consumption of smoked and barbequed meat in our study population is low compared to other parts of the world. Meat is mostly prepared as curries or fried; both of which destroy tissue cysts.

Presence of pet cats in household was not significantly associated with the seropositivity of pregnant women in this study. Similar results were shown by studies conducted in Sri Lanka and overseas [12, 17]. The association of cats and toxoplasmosis is difficult to assess by epidemiological surveys because frequent exposure to cat faeces or lack of preventive measures may enhance the risk of transmission of toxoplasmosis. In Sri Lanka cats do not stay indoors and are allowed to roam freely, thus contaminating the environment heavily. A study conducted in Sri Lanka revealed 30.2% of cats examined were infected with *T. gondii* indicating high level of transmission among definitive hosts [30].

The current study identified household gardening as a significant risk factor associated with *Toxoplasma* IgG seropositivity. The majority of our study population were unemployed thus had time for recreational activities including gardening. Under favourable environmental conditions oocysts can remain infective for many years [31]. Thus the soil can be considered as a potential source for infection in humans. Household gardening involves direct contact with soil contaminated by cat faeces. This explains why household gardening is a significant risk factor for having positive serology for *T. gondii* IgG. Protective gear such as gloves are seldom used hence the infective oocysts in the soil are accidentally ingested by humans during gardening. Contact with soil as is the case with gardening was found to be highly significant in other parts of the world as well for seropositivity of *T. gondii* [26].

## Conclusion

Our study revealed a seroprevalence of 29.9% among pregnant women in the Central Province. This value is higher than other studies done in Sri Lanka indicating a greater level of *T. gondii* transmission in the central highlands of the country. 70.1% were however seronegative and had a risk of primary acute infection during pregnancy which can have adverse effects on the foetus. Thorough screening of high risk groups by early ultrasound scanning and health education to reduce exposure to risk factors by means of simple practical measures involving lifestyle, food preparation and hygiene are practical implementations that could be done based on our findings.

## Abbreviations

ELISA: Enzyme linked immunosorbent assay
PCR: Polymerase chain reaction

## References

[1] Chandrasena N, Herath R, Rupasinghe N, Samarasinghe B, Samaranayake H, Kastuririratne A, de Silva NR. Toxoplasmosis awareness, seroprevalence and risk behavior among pregnant women in the Gampaha district, Sri Lanka. Pathog Glob Health. 2016; 10.1080/20477724.2016.1173325. 10.1080/20477724.2016.1173325 PMC4894262 27092763

[2] Halonen SK, Weiss LMTOXOPLASMOSIS (2013). Handb Clin Neurol.

[3] Derouin F, Pelloux H (2008). Prevention of toxoplasmosis in transplant patients. Clin Microbiol Infect.

[4] Montoya JG, Liesenfeld O (2004). Toxoplasmosis. Lancet.

[5] Montoya JG, Rosso F (2005). Diagnosis and management of toxoplasmosis. Clin Perinatol.

[6] Li XL, Wei HX, Zhang H, Peng HJ, Lindsay DSA (2014). Meta analysis on risks of adverse pregnancy outcomes in *Toxoplasma gondii* infection. PLoS One.

[7] Andiappan H, Nissapatorn V, Sawangjaroen N, Chemoh W, Lau YL, Kumar T, Onichandran S, Suwanrath C, Chandeying V (2014). *Toxoplasma* infection in pregnant women: a current status in Songklanagarind hospital, southern Thailand. Parasit Vectors.

[8] Roizen N, Swisher CN, Stein MA, Hopkins J, Boyer KM, Holfels E, Mets MB, Stein L, Patel D, Meier P, Withers S, Remington J, Mack D, Heydemann PT, Patton D, McLeod R (1995). Neurologic and developmental outcome in treated congenital toxoplasmosis. Paediatrics.

[9] Robert-Gangneux F, Dardé ML (2012). Epidemiology of and diagnostic strategies for toxoplasmosis. Clin Microbiol Rev.

[10] Flegr J, Prandota J, Sovičková M, Israili ZH (2014). Toxoplasmosis – a global threat. Correlation of latent toxoplasmosis with specific disease burden in a set of 88 countries. PLoS One.

[11] Borkakoty B, Biswas D, Jakharia A, Mahanta J (2016). Seroprevalence of *Toxoplasma gondii* among pregnant women in Northeast India. J Assoc Physicians India.

[12] Subasinghe SDLP, Karunaweera ND, Kaluarachchi A, Abayaweera CA, Gunatilake MH, Ranawaka J, Jayasundara DMCS, Gunawardena GSA (2011). *Toxoplasma gondii* seroprevalence among two selected groups of pregnant women. Sri Lankan J Infect Dis.

[13] Yan C, Liang LJ, Zheng KY, Zhu XQ (2016). Impact of environmental factors on the emergence, transmission and distribution of *Toxoplasma gondii*. Parasit Vectors.

[14] Climate of Sri Lanka, Department of Meteorology, Sri Lanka. http://www.meteo.gov.lk/index.php?option=com_content&view=article&id=94&Itemid=310&lang=en. Accessed on 12 July 2017.

[15] Taylor MR, Lennon B, Holland CV, Cafferkey M (1997). Community study of *Toxoplasma* antibodies in urban and rural schoolchildren aged 4 to 18 years. Arch Dis Child.

[16] Munoz-Zanzi C, Williams-Nguyen J, Belongia EA (2013). A sero-survey of toxoplasmosis in farm and non-farm children from Wisconsin, United States, 1997–1999. BMC Public Health.

[17] Bóia MN, Carvalho-Costa FA, Sodré FC, Pinto GMT, Amendoeira MRR (2008). Seroprevalence of *Toxoplasma gondii* infection among Indian people living in Iauareté, São Gabriel da Cachoeira, Amazonas, Brazil. Rev Inst Med trop S Paulo.

[18] Aqeely H, Eman K, El-Gayar DPK, Najmi A, Alvi A, Bani I, Mahfouz MS, Abdalla SE, Elhassan IM. Seroepidemiology of *Toxoplasma gondii* amongst pregnant women in Jazan Province, Saudi Arabia. J Trop Med. 2014; 10.1155/2014/913950. 10.1155/2014/913950 PMC4249603 25484905

[19] Nissapatorn V, Leong TH, Lee R, Init-Ithoi, Ibrahim J, Yen TS (2011). Seroepidemiology of toxoplasmosis in renal patients. Southeast Asian J Trop Med Public Health.

[20] Liesenfeld O, Press C, Montoya JG, Gill R, Isaac-Renton JL, Hedman K, Remington JS (1997). False-positive results in immunoglobulin M (IgM) *Toxoplasma* antibody tests and importance of confirmatory testing: the Platelia Toxo IgM test. Clin Microbiol.

[21] Babaie J, Amiri S, Mostafavi E, Hassan N, Lotfi P, Esmaeili Rastaghi AR, Golkar M (2013). Seroprevalence and risk factors for *Toxoplasma gondii* infection among pregnant women in Northeast Iran. Clin Vaccine Immunol.

[22] Frimpong C, Makasa M, Sitali L, Michelo C (2017). Seroprevalence and determinants of toxoplasmosis in pregnant women attending antenatal clinic at the university teaching hospital, Lusaka, Zambia. BMC Infect Dis.

[23] Ahmadpour GR, Ezatpour B, Hadighi R, Oormazdi H, Akhlaghi L, Tabatabaei F, Azami M, Nejad MM, Mahmoudvand H (2017). Seroepidemiology of *Toxoplasma gondii* infection in pregnant women in west Iran: determined by ELISA and PCR analysis. J Parasit Dis.

[24] Dubey JP, Rajapakse RPVJ, Ekanayake DK, Sreekumar C, Lehmann T (2005). Isolation and molecular characterization of *Toxoplasma gondii* from chickens from Sri Lanka. J Parasitol.

[25] Edelhofer R (1994). Prevalence of antibodies against *Toxoplasma gondii* in pigs in Austria -an evaluation of data from 1982 and 1992. Parasitol Res.

[26] Cook AJC, Gilbert RE, Buffolano W, Zufferey J, Petersen E, Jenum PA, Foulon W, Semprini AE, Dunn DT (2000). Sources of *Toxoplasma* infection in pregnant women: European multicentre case-control study. BMJ.

[27] Wam EC, Sama LF, Ali IM, Ebile WA, Aghangu LA, Tume CB (2016). Seroprevalence of *Toxoplasma gondii* IgG and IgM antibodies and associated risk factors in women of child-bearing age in Njinikom, NW Cameroon. BMC Res Notes.

[28] Sakikawa M, Noda S, Hanaoka M, Nakayama H, Hojo S, Kakinoki S, Nakata M, Yasuda T, Ikenoue T, Kojima T (2012). Anti-Toxoplasma antibody prevalence, primary infection rate, and risk factors in a study of toxoplasmosis in 4,466 pregnant women in Japan. Clin Vaccine Immunol.

[29] Flatt A, Shetty N (2013). Seroprevalence and risk factors for toxoplasmosis among antenatal women in London: a re-examination of risk in an ethnically diverse population. Eur J Pub Health.

[30] Kulasena VA, Rajapakse RPVJ, Dubey JP, Dayawansa PN, Premawansa S. Seroprevalence of *Toxoplasma gondii* in cats from Colombo, Sri Lanka. J Parasitol 2011;97(1):152-152. 10.1645/GE-2640.1 21348625

[31] Torrey EF, Yolken RH (2013). *Toxoplasma* oocysts as a public health problem. Trends Parasitol.

